# Culprit versus Complete Revascularization during the Initial Intervention in Patients with Acute Coronary Syndrome Using a Virtual Treatment Planning Tool: Results of a Single-Center Pilot Study

**DOI:** 10.3390/medicina59020270

**Published:** 2023-01-31

**Authors:** Deniss Vasiljevs, Natalja Kakurina, Natalja Pontaga, Baiba Kokina, Vladimirs Osipovs, Nikolajs Sorokins, Sergejs Pikta, Karlis Trusinskis, Aivars Lejnieks

**Affiliations:** 1 Daugavpils Regional Hospital, 20 Vasarnicu Str., LV-5417 Daugavpils, Latvia; 2 Department of Internal Diseases, Riga Stradins University, 16 Dzirciema Str., LV-1007 Riga, Latvia; 3 Latvian Center of Cardiology, Pauls Stradins Clinical University Hospital, 13 Pilsonu Str., LV-1002 Riga, Latvia; 4 Riga East Clinical University Hospital, 2 Hipokrata Str., LV-1038 Riga, Latvia

**Keywords:** multivessel, acute coronary syndrome, instantaneous wave free ratio, virtual percutaneous coronary intervention, iFR, complete revascularization, non-culprit intervention

## Abstract

*Background and Objectives:* The revascularization strategy for percutaneous coronary intervention (PCI) in patients with multivessel (MV) acute coronary syndrome (ACS) remains controversial. Certain gaps in the evidence are related to the optimal timing of non-culprit lesion revascularization and the utility of instantaneous wave-free ratio (iFR) in the management of MV ACS intervention. The major benefits of iFR utilization in MV ACS patients in one-stage complete revascularization are: (1) the possibility to virtually plan the PCI, both the location and the extension of the necessary stenting to achieve the prespecified final hemodynamic result; (2) the opportunity to validate the final hemodynamic result of the PCI, both in culprit artery and all non-culprit arteries and (3) the value of obliviating the uncomfortable, costly, time consuming and sometimes deleterious effects from Adenosine, as there is no requirement for administration. Thus, iFR use fosters the achievement of physiologically appropriate complete revascularization in MV ACS patients during acute hospitalization. *Materials and Methods:* This pilot study was aimed to test the feasibility of a randomized trial research protocol as well as to assess patient safety signals of co-registration iFR-guided one-stage complete revascularization compared with that of standard staged angiography-guided PCI in de novo patients with MV ACS. This was a single-center, prospective, randomized, open-label clinical trial consecutively screening patients with ACS for MV disease. The intervention strategy of interest was iFR-guided physiologically complete one-stage revascularization, in which the virtual PCI planning of non-culprit lesions and the intervention itself were performed in one stage directly following treatment of the culprit lesion and other critical stenosis of more than ninety percent. Seventeen patients were recruited and completed the 3-month follow-up. *Results:* Index PCI duration was significantly longer while the volume of contrast media delivered in index PCI was significantly greater in the iFR-guided group than in the angiography-guided group (119.4 ± 40.7 vs. 47 ± 15.5 min, *p* = 0.004; and 360 ± 97.9 vs. 192.5 ± 52.8 mL, *p* = 0.003). There were no significant differences in PCI-related major adverse cardiovascular events (MACE) between the groups during acute hospitalization and at 3-months follow-up. One-stage iFR-guided PCI requires fewer PCI attempts until complete revascularization than does angiography-guided staged PCI. *Conclusions:* Complete revascularization with the routine use of the virtual planning tool in one-stage iFR-guided PCI is a feasible practical strategy in an everyday Cath lab environment following the protocol designed for the study. No statistically significant safety signals were documented in the number of PCI related MACE during the 3-month follow-up.

## 1. Introduction

Approximately 40% of patients with ST-segment elevation myocardial infarction (STEMI) and up to 70% of patients with non-ST-segment elevation myocardial infarction (NSTEMI) have significant multivessel (MV) coronary artery disease (CAD), indicating the presence of significant lesions in addition to the culprit lesion [1,2,3]. This patient group is associated with higher risks and worse clinical outcomes of acute coronary syndrome (ACS), requiring more complex management approaches and percutaneous coronary intervention (PCI) strategies than do patients with single-vessel coronary artery disease [4]. Complete revascularization for MV CAD, including non-culprit lesion treatment, potentially improves prognosis. Nevertheless, this strategy has varying success rates under different clinical settings [5,6,7,8]. Previous studies have reported that complete MV revascularization tends to have positive outcomes [9,10,11]. The main study design mentioned in this paper aims to address two aspects: 1: the utility of instantaneous wave-free ratio (iFR) in the management of non-culprit disease; and 2: the optimal timing of non-culprit intervention. Whether simultaneous complete revascularization is superior to staged approaches for index PCI remains controversial due to the current ambiguous data, which suggest integrated and individualized approaches [12,13,14,15]. In this regard, myocardial ischemia evaluation has been highlighted [16], and physiology-guided coronary revascularization, including advanced invasive functional assessment tools, is of great clinical interest [17,18]. Instantaneous wave-free ratio co-registration software, with potential virtual PCI planning, is an option for optimizing the decision-making processes. This method is innovative, promising, and informative for the evaluation of hemodynamic significance of coronary lesions [19,20], including MV CAD [21]. However, the therapeutic implications of iFR for assessing non-culprit coronary plaques in patients with ACS requires further research to obtain a more convincing conclusion [22]. This issue is also highlighted in the current European Society of Cardiology Guidelines on myocardial revascularization [8]. This pilot study aimed to test the feasibility of a randomized trial research protocol and to highlight preliminary data on the safety of iFR co-registration advanced guidance for physiologically complete one-stage revascularization, and compare the findings with those for staged angiography-guided complete revascularization by PCI in patients with ACS combined with MV CAD.

## 2. Materials and Methods

### 2.1. Study Population

This was a single-center, prospective, randomized, open-label clinical trial consecutively screening patients with ACS for MV disease. The study population included patients with sinus rhythm only, to precisely measure left chamber segmental myocardial deformation and peak mechanical dispersion. Simple randomization method has been used without cross-over between groups. Seventeen patients were initially enrolled in this study and completed the 3-month follow-up during the period of two years (2020–2021). Trial performed in Cardiology and internal diseases center of Daugavpils Regional Hospital, Latvia. First round patient selection was performed by intensive care unit and emergency department clinicians. Where NSTEMI patients received echocardiography prior to the acute coronary angiography. And STEMI patients received echocardiographic evaluation in the first 24 h after acute coronary angiography to re-check for exclusion criteria.

### 2.2. Inclusion and Exclusion Criteria

**Figure 1:**
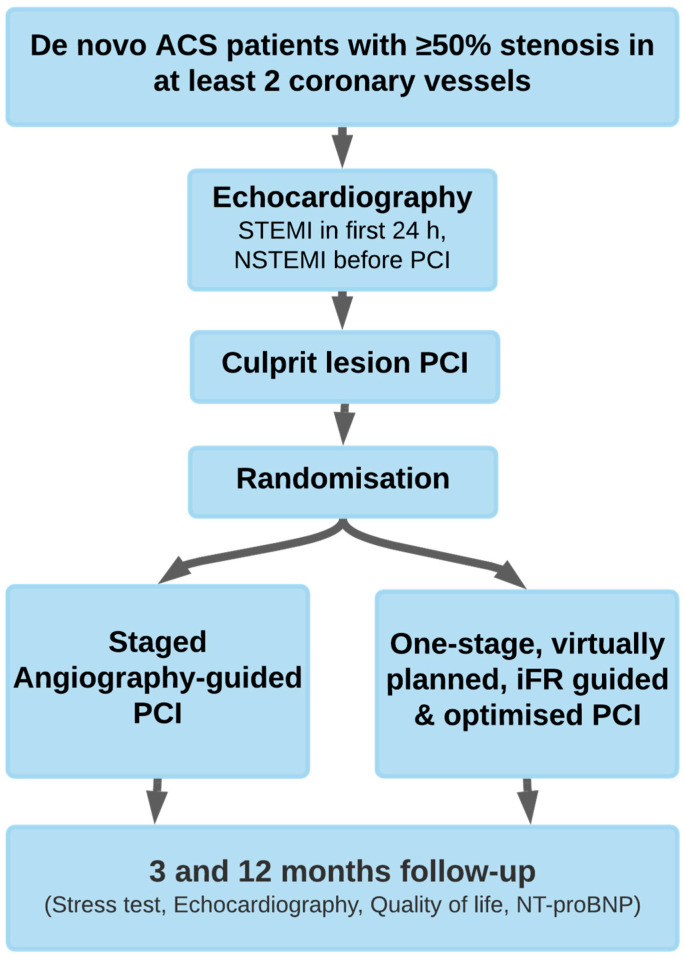
Trial design. ACS = Acute coronary syndrome; STEMI = ST-Elevation myocardial infarction; NSTEMI = non–ST-segment elevation myocardial Infarction; PCI = Percutaneous coronary intervention; iFR = instantaneous wave-free ratio; NT-proBNP = N-terminal pro-brain natriuretic peptide.

The ‘all-comers’ study population comprised hemodynamically stable de novo patients with ACS and a stenosis of ≥50% by visual estimation in at least two separate coronary arteries, as well as a Thrombolysis in myocardial infarction flow ≥2 (Figure 1). All patients provided written informed consent for participation.

Acute coronary syndrome diagnosis was confirmed using angina pectoris symptoms, electrocardiography changes, and/or hs-troponin I levels above the reference range. Exclusion criteria were inability to give consent, age <18 years, significant valve disease requiring surgical intervention, cardiogenic shock, reduced kidney function (estimated glomerular filtration rate <30 mL/min/1.73 m^2^), congenital heart defects, acute pulmonary artery embolism, isolated left main ostial stenosis, active oncologic treatment, toxic cardiomyopathy, and inability to perform revascularization by PCI due to comorbidities or patient refusal. The design of this trial required the mandatory use of drug-eluting stents in both treatment groups.

### 2.3. Instantaneous Wave-Free Ratio Measurements

**Figure 2:**
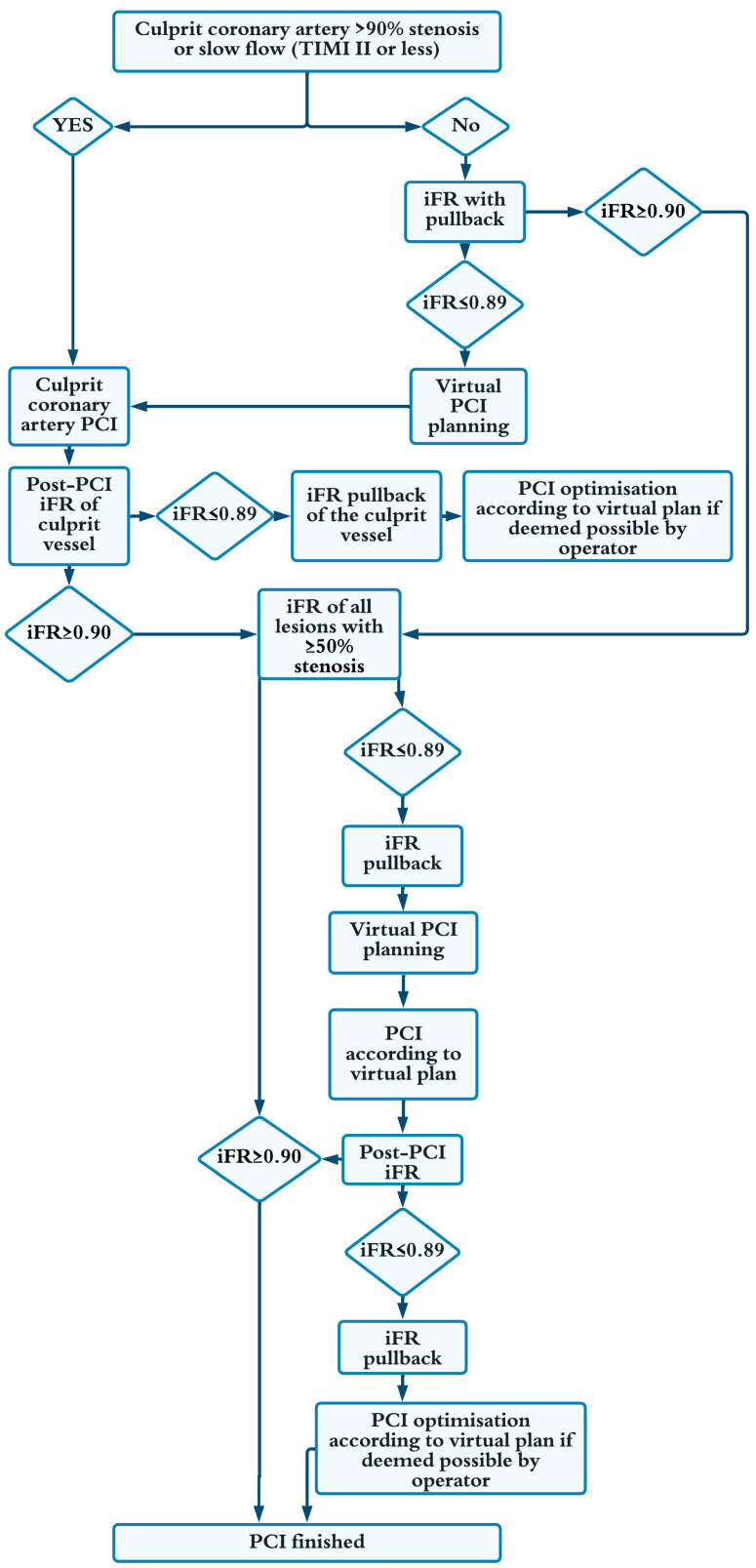
iFR-guided and optimised group intervention protocol. TIMI = thrombolysis in myocardial infarction flow; iFR = instantaneous wave-free ratio; PCI = percutaneous coronary intervention.

The intervention strategy of interest was iFR-guided physiologically complete one-stage revascularization, in which the virtual PCI planning of non-culprit lesions and the intervention itself were performed in one stage directly following treatment of the culprit lesion and other critical stenosis of more than ninety percent (Figure 2). Patients with chronic total occlusions in both groups were allowed to undergo staged PCI.

iFR is an invasive diagnostic tool produced by Philips, with co-registration tools available via SyncVision software (version 4.1.0.5, Philips Volcano, Zaventem, Belgium). In iFR technology, a wire-based pressure transducer measures the aortic and coronary pressures distal to the stenosis in an isolated period during diastole when the relationship between pressure and flow is linear and constant. iFR is subsequently calculated. After the distal point iFR is calculated based on five heartbeats, the pullback co-registration with the last angiogram is obtained. During pullback, a pressure wire is slowly withdrawn under fluoroscopic control to the tip of the guiding catheter, measuring the ratio of every millimeter. Following pullback, the software transposes the ratio results to the results of the last angiogram, allowing the operator to directly observe pressure drops in the coronary artery. This allows virtual PCI planning, which in turn allows the operator to see the final estimated results of distal iFR by placing a virtual stent in the SyncVision software. Patients with an iFR ≤0.89 were considered severely ischemic and were recommended to undergo PCI treatment. The mandatory PCI result evaluation by iFR was performed. In patients with a post-PCI iFR ≤0.89 further PCI optimization was recommended, if feasible. Further optimization feasibility was determined based on information from post-PCI pullback co-registration. The concept of iFR is like that of fractional flow reserve (FFR), but it is easier to perform and provokes less patient discomfort since hyperemic agents are not needed. Moreover, iFR allows PCI planning based on pullback co-registration results as multiple lesions do not interfere with result calculations. In this study, the patients treated using virtual PCI planning by iFR co-registration were assigned to the iFR-guided group.

### 2.4. Primary Safety Endpoints

The primary safety endpoints of this study were PCI-related major adverse cardiovascular events (MACE), comprising cardiac death, non-fatal myocardial infarction (MI), unplanned revascularization, stent thrombosis, flow limiting coronary dissections, no-reflow phenomenon, coronary perforations, coronary distal embolization, vascular access site complications requiring blood transfusion or surgical intervention, and contrast-induced nephropathy requiring hospitalization. Non-fatal MI was defined by criteria for acute myocardial infarction by the Fourth universal definition of myocardial infarction. The study was conducted in accordance with the ethical principles of the Declaration of Helsinki and approved by the Ethics Committee of the Riga Stradins University. The ClinicalTrials.gov identifier is NCT05006183.

### 2.5. Statistical Analysis

IBM SPSS^®^ Statistics for Windows, version 22.0 (IBM Corp., Armonk, NY, USA) was used for all statistical analyses. Patient characteristics were analyzed using descriptive statistical methods, with mean values as central tendency indicators and standard deviations as dispersion indicators. If the data were not normally distributed, median values and interquartile ranges were used. Data compliance with normal distribution was determined by the Shapiro-Wilk test. In case of normally distributed continuous data “*t*-test” was used to compare means of two groups. According to the number of patients in both groups, Fisher’s exact test was used to compare qualitative nominal variables, and the nonparametric Mann-Whitney U-test was used to compare quantitative linear variables in two samples. Statistical power is determined by the following variables: alpha cut-off of 5%, beta cut-off of 20%, power of 80%, iFR-guided group incidence of 1.8% and angiography-guided group incidence of 18.3% [23,24]. A sample size of 51 patients per group was calculated with total of 102 patients needed to include in the main trial.

## 3. Results

Of patients included in the trial, seven (41.2%) were female, and 10 (58.8%) were male, with a median age of 64 years and median body mass index of 28.5 ± 4.6 kg/m^2^. There were no significant differences in baseline characteristics between the groups, except for age, as patients in the angiography-guided group were significantly older than were those in the iFR-guided group (mean, 62.7 ± 4.5 vs. 69.6 ± 8.2 years, *p* = 0.046) (Table 1).

During acute hospitalization, there were no statistically significant differences in the amount and length of stents implanted, the length of hospital stay, and the number of PCI-related MACEs between groups. Index PCI duration was significantly longer, and the volume of contrast media delivered was significantly greater in the iFR-guided group than in the angiography-guided group (119.4 ± 40.7 vs. 47.0 ± 15.5 min, *p* = 0.004; and 360.0 ± 97.9 vs. 192.5 ± 52.8 mL, *p* = 0.003) (Table 2).

Though when complete myocardial revascularization was achieved, in both groups (results collected at the 3-month follow-up) there were no significant differences in: the amount and length of stents implanted; the total volume of contrast media delivered; total PCI duration; the length of overall hospital stay; and total PCI-related MACEs (comprising of cardiac death, non-fatal myocardial infarction (MI), unplanned revascularization, stent thrombosis, flow limiting coronary dissections, no-reflow phenomenon, coronary perforations, coronary distal embolization, vascular access site complications requiring blood transfusion or surgical intervention, and contrast-induced nephropathy requiring hospitalization) between groups. The complete simultaneous myocardial revascularization strategy executed in the iFR-guided group required a significantly smaller number of PCI attempts than did the staged approach of the angiography-guided group (1.0 ± 0.0 vs. 1.8 ± 0.7 PCIs, *p* = 0.007) (Table 2). Four interventional cardiologists were involved in the trial and there were no cross-over between groups. The initial trial protocol design showed to be practical and feasible to accomplish in an everyday Cath lab environment.

## 4. Discussion

In this pilot study, the iFR-guided and virtual planning tool-optimized PCI strategy was tested for safety and feasibility as a revascularization strategy in patients with MV ACS. Despite its significantly longer procedure and higher volume of the contrast medium delivered at the index PCI, there were no significant differences in safety endpoints (PCI-related MACEs comprising of cardiac death, non-fatal myocardial infarction (MI), unplanned revascularization, stent thrombosis, flow limiting coronary dissections, no-reflow phenomenon, coronary perforations, coronary distal embolization, vascular access site complications requiring blood transfusion or surgical intervention, and contrast-induced nephropathy requiring hospitalization) between the angiography and iFR-guided groups at the 3-month follow-up. Furthermore, except for two cases of distal embolization in the iFR group, and one case of flow-limiting coronary dissection in the angiography-guided group during the second revascularization stage, there were no cardiovascular deaths or non-fatal MIs, and no patients needed unplanned revascularization. Moreover, there were no cases of no-reflow phenomenon, coronary perforation, stent thrombosis, contrast-induced nephropathy requiring hospitalization, and vascular access site complications requiring blood transfusion or surgical intervention.

Overall, using invasive physiological coronary blood flow indices to assess lesion functional significance improves clinical outcomes in patients with MV CAD [25] and in patients with non-culprit coronary lesions during the acute phase [26,27]. FFR has been widely investigated, and after obtaining significant data regarding a reduction in the composite endpoint, which includes death, MI, and repeat revascularization, in the Fractional Flow Reserve versus Angiography for Multivessel Evaluation (FAME) study, evidence on the utility of FFR for the management of patients with ACS began to emerge [24]. Complete FFR-guided revascularization during index hospitalization for patients with STEMI significantly reduced primary endpoints, including mortality, MI, revascularization, and cerebrovascular events in the Compare-Acute [28] trial and all-cause mortality, non-fatal reinfarction, and ischemia-driven revascularization in the Third DANish Study of Optimal Acute Treatment of Patients With STEMI: PRImary PCI in MULTIvessel Disease (DANAMI-3-PRIMULTI) trial [29]. Patients with NSTEMI and MV CAD were enrolled in the Fractional flow reserve versus Angiography in guiding Management to Optimise oUtcomeS in Non-ST-segment Elevation Myocardial Infarction (FAMOUS-NSTEMI) trial, in which FFR-guided management was compared with angiography-only evaluation. There were significant differences in the reduction rate of revascularizations, but not the health outcomes and quality of life, between these methods [30]. However, the published results of the FLOW Evaluation to Guide Revascularization in Multi-vessel ST-elevation Myocardial Infarction (FLOWER-MI) trial are less encouraging than those of the aforementioned trial as they indicated that there were no significant differences in the risk of death, MI, and urgent revascularization [31]. Nevertheless, it is important to note that the FLOWER-MI trial only included STEMI patients and FFR-guided revascularization was performed, whereas in our study, patients with STEMI and those with NSTEMI were enrolled and assigned to the iFR group.

Both FFR and iFR methods showed comparable results and diagnostic accuracy for non-culprit lesion assessment in the WAVE study in patients with STEMI [32] and in the iFR-SWEDEHEART study in patients with NSTEMI [33]. While FFR measurements are conducted under hyperemic settings, iFR measurements are based on the resting pressure gradient during a specific phase of diastole and are less uncomfortable than are FFR measurements for patients [34,35,36]. Furthermore, complex flow interaction in tandem stenoses under hyperemic conditions may induce incorrect MV CAD-related measurements; however, iFR may optimize such evaluations [37]. Data from post-PCI coronary physiology evaluations in the DEFINE PCI study revealed that almost every fourth patient had significant residual ischemia following successful angiography-guided revascularization, highlighting the diagnostic value of iFR [38]. Our research provides additional essential data and questions the existing evidence. Nevertheless, we believe that more results of expanded patient groups are warranted, evaluating longer-term outcomes and procedural benefit-related endpoints.

Regarding the treatment of non-culprit arteries, the currently available evidence indicates that complete revascularization PCI is better than culprit-only revascularization for MV CAD. The COMPLETE trial demonstrated that complete revascularization for STEMI significantly reduced coprimary outcomes, including cardiovascular death, MI, and ischemia-driven revascularization [39]. These findings support data from the PRAMI trial, in which the primary composite outcome of death from cardiac causes, non-fatal MI, or refractory angina was significantly reduced in patients with STEMI who underwent preventive PCI for non-culprit lesions [40]. Rathod et al. also demonstrated in NSTEMI patients with MV disease that although in the long term it leads to a decrease in mortality, in the acute period higher mortality rates were observed in those with complete revascularization, compared to those with revascularization of only the culprit lesion [41]. The need for further research regarding this matter is highlighted, and non-culprit lesion treatment during index revascularization for ACS remains controversial according to data summarized in published meta-analyses [42,43]. Nevertheless, the SMILE trial supported the fact that, compared with other treatment methods, one-stage complete revascularization is the optimal strategy for patients with NSTEMI and MV disease, with significantly reduced major adverse cardiovascular and cerebrovascular events, including death, stroke, and MI [44]. There is still no consensus in meta-analyses on whether one-stage complete revascularization should be used for patients with STEMI [13,40,42], although recent data have been more supportive of the single-stage approach than have previous data [28,43]. Specifically, in the CvLPRIT trial, compared with other approaches, index admission complete coronary revascularization tended to have positive outcomes in patients with STEMI, with significantly reduced primary endpoints, including major adverse cardiac events, mortality, recurrent MI, heart failure, and repeat revascularization [45].

In this study angiography-guided staged PCI strategy had a tendency towards longer overall hospital stay needed for complete revascularization. Additionally, iFR-guided PCI enabled complete revascularization in a single stage, demonstrating a significantly reduced number of PCIs needed in achievement of complete revascularization. This indicates several potential benefits in terms of the overall PCI-related risk since each procedure is associated with potential risks of vessel dissection, perforation, and hemodynamic complications [46]. Thus, the iFR-guided strategy may benefit the temporal dilution of procedural complications. Moreover, due to the COVID-19 pandemic, shorter or one-time hospitalizations may be safer and beneficial for both patients and the healthcare system.

## 5. Conclusions

Complete revascularization with the routine use of the virtual planning tool in one-stage iFR-guided PCI is a feasible practical strategy in an everyday Cath lab environment following the protocol designed for the study. One-stage iFR-guided PCI requires fewer PCI attempts until complete revascularization than does angiography-guided staged PCI. No statistically significant safety signals were documented in the number of PCI related MACE both during acute hospitalization and at 3-months follow-up. Further studies will help assess the role of routine iFR co-registration guidance in one-stage complete revascularization PCI to treat patients with MV ACS. After the lack of safety signals in the pilot study, further trial progress commenced to gain statistical power.

## Figures and Tables

**Table 1:** Baseline characteristics of patients undergoing one-stage instantaneous wave-free ratio-guided and staged angiography-guided percutaneous coronary intervention.

Baseline Variable	iFR-Guided Group (*n* = 9)	Angiography-Guided Group (*n* = 8)	*p*-Value
NSTEMI + UA	5 (55.6%)	7 (87.5%)	0.294
STEMI	4 (44.4%)	1 (12.5%)	0.294
Female	2 (22.2%)	5 (62.5%)	0.153
Male	7 (77.8%)	3 (37.5%)	0.153
Age, mean years	62.7 ± 4.5	69.6 ± 8.2	0.046
BMI, mean (kg/m^2^)	26.1 ± 4.8	29.3 ± 4.1	0.236
Smokers	5 (55.6%)	4 (50.0%)	0.637
Pack-years, mean	25.4 ± 19.7	20.6 ± 23.1	0.727
History of hypertension	5 (55.6%)	7 (87.5%)	0.294
History of diabetes mellitus	0	3 (37.5%)	0.082
History of congestive heart failure	6 (66.7%)	3 (37.5%)	0.347
NT-proBNP, mean (pg/mL)	1270 ± 800.8	2054 ± 1721.6	0.386
Creatinine, mean (µmol/L)	73.7 ± 22.3	86 ± 26.6	0.148
Hgb, mean (g/dL)	13 ± 1.1	12 ± 2.4	0.643
HbA1c, mean (%)	5.86 ± 0.5	7 ± 1.8	0.051
Peak index hs-troponin I (pg/mL)	2227 ± 1486.8	3886 ± 3816.9	0.685
LDL cholesterol, mean (mmol/L)	4.13 ± 1.3	3.42 ± 1.5	0.290
Total cholesterol, mean (mmol/L)	5.28 ± 1.3	5.55 ± 0.7	0.558
HDL cholesterol, mean (mmol/L)	1.05 ± 0.3	1.14 ± 0.3	0.773
Index EF, mean (%)	51.9 ± 19.9	57.4 ± 11.4	0.314

BMI = body mass index; EF = ejection fraction; HbA1c = haemoglobin A1c; HDL = high-density lipoprotein; Hgb = haemoglobin; iFR = instantaneous wave-free ratio; LDL = low-density lipoprotein; NSTEMI = non–ST-segment elevation myocardial infarction; NT-proBNP = N-terminal pro-brain natriuretic peptide; STEMI = ST-elevation myocardial infarction; UA = unstable angina.

**Table 2:** Index procedure and complete coronary revascularization outcomes of patients undergoing complete revascularization in one-stage iFR-guided PCI versus staged angiography-guided PCI at the 3-month follow-up.

Procedural Outcomes	Angiography-Guided Group (*n* = 8)	iFR-Guided Group (*n* = 9)	*p*-Value
Index PCI stent length used (mm)	39.9 ± 19	55.2 ± 31	0.335
Index PCI Number of stents used	1.5 ± 0.5	1.89 ± 1.1	0.526
Index PCI volume of contrast media (mL)	192.5 ± 52.8	360 ± 97.9	0.003
Index PCI duration (minutes)	47 ± 15.5	119.4 ± 40.7	0.004
Index hospital stay (days)	5 ± 1.6	4.8 ± 1.1	0.878
Index PCI related MACE	Median = 0.0 IQR = 0.0–0.0	Median = 0.0 IQR = 0.0–0.5	0.168
Total stent length used (mm)	67.6 ± 42.7	55.2 ± 31	0.441
Total number of stents used for complete revascularization	2.8 ± 1.3	1.9 ± 1.1	0.134
Total volume of contrast media (mL)	313.8 ± 164.4	360 ± 97.9	0.563
Total PCI duration (minutes)	101 ± 55	119 ± 40	0.563
Total hospital stay (days)	6.5 ± 2.3	4.8 ± 1.1	0.058
Total PCI number till complete revascularization achieved	1.8 ± 0.7	1.0 ± 0.0	0.007
Total PCI related MACE	Median = 0.0 IQR = 0.0–0.0	Median = 0.0 IQR = 0.0–0.5	0.611

MACE = major adverse cardiovascular events; PCI = percutaneous coronary intervention.

## Data Availability

Not applicable.
